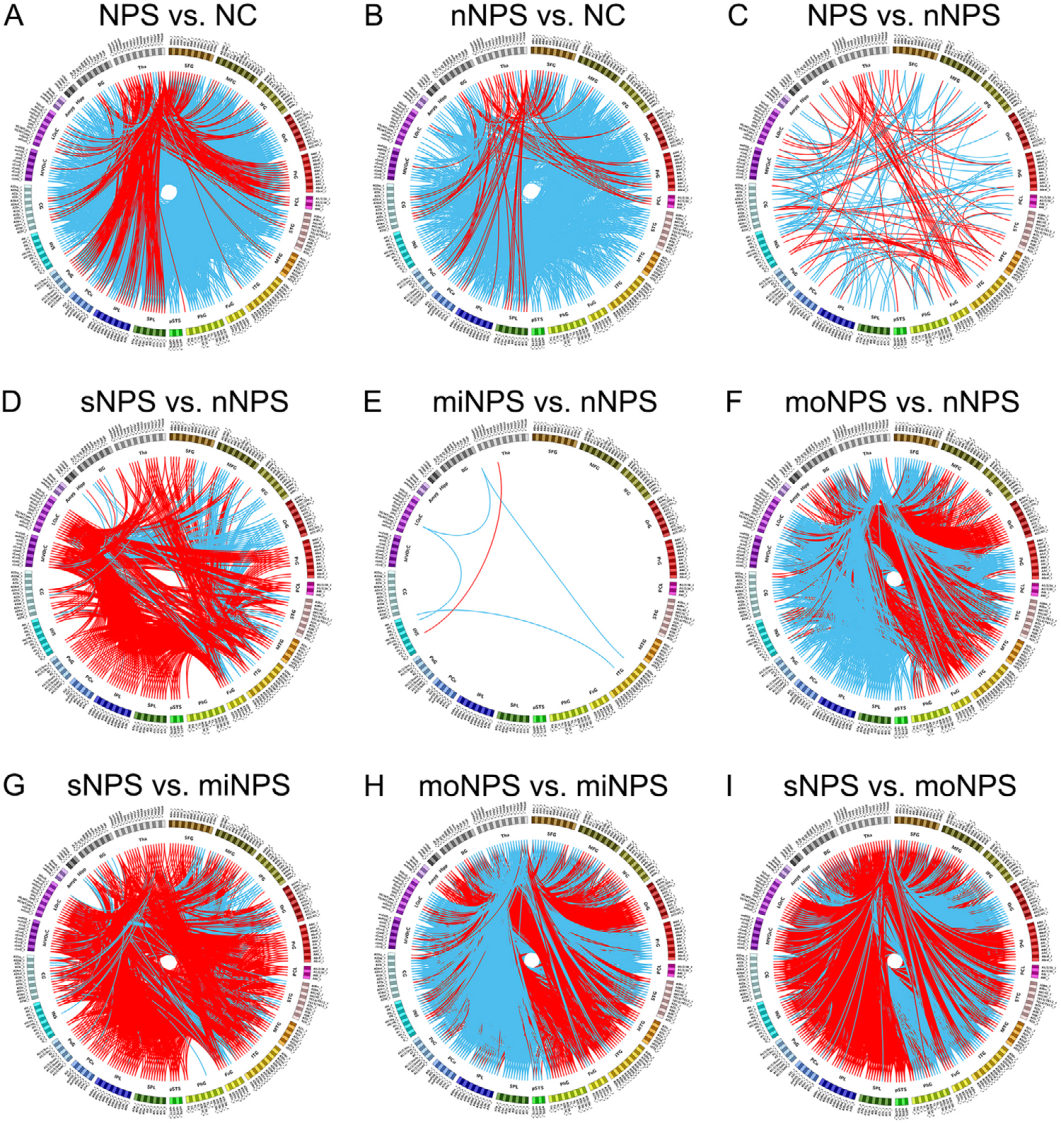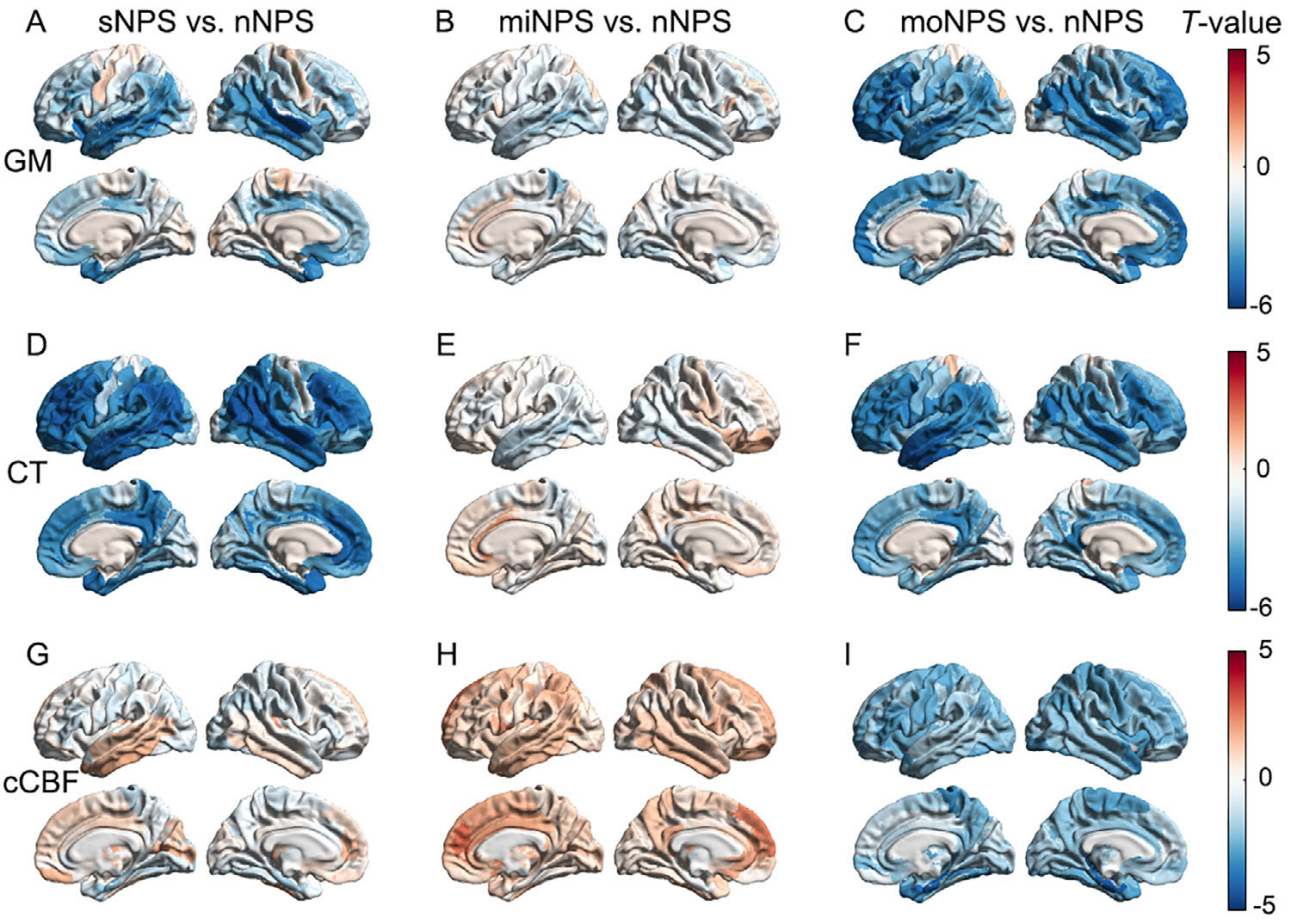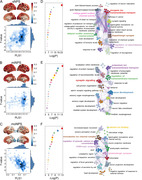# Macroscale structural covariance network reveals the biological mechanisms of neuropsychiatric symptoms in the Alzheimer’s continuum

**DOI:** 10.1002/alz.084816

**Published:** 2025-01-03

**Authors:** Jiwei Jiang, Kun Zhao, Peiyang Zheng, Shirui Jiang, Jun Xu

**Affiliations:** ^1^ Beijing Tiantan Hospital, Capital Medical University, Beijing China; ^2^ School of Artificial Intelligence, Beijing University of Posts and Telecommunications, Beijing China; ^3^ Beijing Tiantan Hospital, Capital Medical University, Beijing China

## Abstract

**Background:**

The intricate and heterogeneous phenotypes associated with neuropsychiatric symptoms (NPSs) encumber exploration of their role in the neuropathology and underlying biological mechanisms of Alzheimer’s disease (AD) continuum.

**Method:**

An individual‐level Regional Radiomics Similarity Network (R2SN) for 487 patients with AD continuum (376 with NPSs vs. 111 without NPSs) were developed to find the R2SN connections associated with NPSs and refine the subtypes of NPS in the AD continuum. Distinct brain network dysfunction, multimodal neuroimaging burden, and clinical measures/progression of each NPS subtype were analyzed. Gene set enrichment analysis (GSEA) was used to explore the biological mechanisms underpinning the various NPS subtypes and their connections to the biological pathways leading to the AD continuum.

**Result:**

Three NPS subtypes were identified based on 300 distinct key R2SN connections. Compared to AD patients without NPS, the first NPS subtype (sNPS) and third NPS subtype (moNPS) exhibited significant opposite pattern of brain connectivity damage, while the second NPS subtype (miNPS) showed minimal differences (Figure 1). Moreover, both sNPS and moNPS subtypes exhibited diminished performance at baseline and rapid decline in the MMSE and MoCA scores, while no statistically significant difference was discerned between the miNPS subtype and those without NPS. Furthermore, both sNPS and moNPS subtypes exhibited reduced regional grey matter volume and cortical thickness in the frontal, temporal, and parietal lobules, while miNPS subtype lacks significant structural brain changes but exhibited higher corrected cerebral blood flow in the inferior frontal gyrus and anterior cingulate cortex, suggesting early compensatory cerebral hyperperfusion (Figure 2). GSEA unveiled the shared and subtype‐specific gene pathways, elucidating each subtype’s unique biological mechanisms associated with the contribution of NPS‐related specific brain connectome dysfunctions to AD progression (Figure 3).

**Conclusion:**

This is the first study to identify three distinct NPS subtypes in the AD continuum through a data‐driven approach, bridging the gap in the knowledge on the contributions of NPSs to the onset and development of AD. Our study constitutes a step toward resolving stagnant research progress on NPS management strategies based on clinical symptoms, and offers new insights into precise interventions for these patients.